# Intra-Cranial Tumour: A Clinical Record and Commentary

**Published:** 1904-06-04

**Authors:** C. O. Hawthorne

**Affiliations:** Physician to the Central London Ophthalmic Hospital, Assistant Physician to the North-West London and Royal Waterloo Hospitals.


### HOSPITAL CLINICS.

### INTRA-CRANIAL TUMOUR: A CLINICAL RECORD AND COMMENTARY.

By C. O. HAWTHORNE, M.D., M.R.C.P., Physician to the Central London Ophthalmic Hospital, Assistant Physician to the North-West London and Royal Waterloo Hospitals.

DOUBLE OPTIC NEURITIS, WITH OCCASIONAL HEADACHE AND VOMITING; CESSATION OF SYMPTOMS, EXCEPT DEFECT OF VISION FROM OPTIC ATROPHY, FOR THREE YEARS; THEN RATHER RAPID UNCONSCIOUSNESS AND DEATH.

THE patient of whose case the above is a brief summary first came under observation in January 1901. He was then a youth of 15 years, and save for a very serious defect of vision professed himself to be in good and even vigorous health. Both his general appearance and a careful physical examination supported this statement. No evidence of disease could be found in the thoracic or abdominal viscera, and the only noteworthy feature observed in examining the nervous apparatus was a somewhat marked character of the knee-jerks. The ophthalmoscope showed each optic disc to be very white, with narrowed arteries and some evidences of exudation along the retinal vessels. The appearances, in short, were those of a post-neuritic optic atrophy and readily accounted for the fact that the boy could barely manage to count fingers at a distance of 3 feet. Thus, in January 1901, the patient was practically blind from post-neuritic optic atrophy, but otherwise was entirely free from all signs and symptoms of disease. In his view, indeed, it was this defect of vision which constituted his disease, and he would hardly admit that he had ever had much other ground for complaint. According to his story, his vision had been quite good until June 1900, when it somewhat suddenly failed, and it has remained practically without further change until the present date. On questioning the boy, however, he admits that on several occasions previous to the failure of vision he had rather severe headache and with this some vomiting. But only on a single day did he remain in his room on account of these symptoms; otherwise he went to school as usual, though on a number of days he was unable to work, and the schoolmaster sent him home. Considering these facts, it is evident that the patient early in 1900 suffered from some condition which produced double optic neuritis with attacks of headache and some vomiting. At first the vision was not affected by the neuritis, as indeed is often the case. It is not during the stage of inflammatory exudation that vision suffers, but rather when the exudation undergoes organisation, and pressure is thus produced on the optic nerve fibres. There can be no doubt that had the boy been examined with the ophthalmoscope at the time he was having occasional headaches and attacks of vomiting he would have been found to be the subject of double optic neuritis. The vomiting and headache were comparatively slight, yet they were manifestly symptoms of possibly cerebral origin, and in such circumstances the use of the ophthalmoscope is imperative. That sight is not defective matters not; the most intense optic neuritis is quite compatible with perfect vision. And, as is evident from the present case, an event or condition competent to produce a severe optic neuritis, with subsequent atrophy and practical blindness, may fail to give any other evidences of its activity beyond an occasional headache or attack of vomiting. The question next demanding consideration is the nature of the event or condition, of which the three symptoms—double optic neuritis, headache, and vomiting—were expressions. It is no doubt possible to have such symptoms as a result of abnormal blood conditions, such for example as that due to kidney disease or (occasionally) chlorosis. But the first presumption arising from a clinical record which includes optic neuritis, headache, and vomiting is that of some serious intra-cranial lesion, and as the patient presented no evidence of renal or other form of disease it seems necessary to accept this presumption as the first step in the construction of the diagnosis. Now the intra-cranial events capable of producing double optic neuritis are not numerous. Possibly, as a very exceptional fact, sinus thrombosis ought to be included; but reserving this, cerebral tumour, meningitis, and cerebral abscess are the three conditions which demand consideration when a patient is found to be the subject of double optic neuritis. Now in the present instance abscess may certainly be excluded, for the boy had never had any form of ear disease, and he recovered so far as his headache and vomiting were concerned without the operative interference which an abscess requires. Meningitis, at least in any of its usually recognised varieties, can hardly be attached as a diagnosis to a patient who has never been confined to bed even for a single day, and who denies that he has even at any time been seriously ill. There remains therefore tumour; and, other reasons apart, there is one positive ophthalmoscopic fact in favour of this view. This is the extreme degree of optic nerve atrophy. From this it may safely be concluded that the optic neuritis was extreme. And whilst many conditions may cause optic neuritis it is certain that this in its most extreme form is mainly seen in cases of intra-cranial tumour. Against the diagnosis of tumour some may be disposed to urge the recovery (except the blindness) of the patient. But many facts and experiences could be quoted in favour of the view that an intra-cranial tumour may cease to grow, and that the symptoms produced by such a tumour may entirely subside. More than one patient, after an illness followed by blindness, has lived in good general health for many years, and after death a shrunken and atrophied tumour has been found in the brain. The present case may fairly be regarded as a parallel to such records. Unfortunately the parallel is incomplete in respect to the fact that so far from living for many years and dying from some other disease, the present patient died within three years of his first attack, and obviously from some reawakened activity of the cerebral condition responsible for his earlier symptoms. Such an experience is not merely compatible with a diagnosis of tumour; it lends support to that diagnosis. So long as a tumour is growing it produces congestion and irritation in its neighbourhood, and these conditions, rather than mere mechanical pressure and displacement, are directly responsible for the clinical disturbances which indicate the existence of an intra-cranial tumour. Should the tumour cease to grow, the neighbouring irritation and congestion, and the symptoms that depend upon them, subside. But if, as is always possible, a quiescent tumour again becomes active, a repetition of the consequences of such growth will necessarily take place. There can be little doubt that this in essence was the pathological movement in the case which is here under discussion. In 1900, it may be assumed, the boy was the victim of a newly developing intra-cranial tumour which produced severe optic neuritis with some headache and vomiting; after six months or so the tumour became quiescent and the general symptoms subsided, but optic atrophy was caused as the neuritis subsided; for three years or so the intra-cranial tumour made no sign, but at the end of that time it again became active and produced severe headache with rapidly developing coma and death. The absence of a post-mortem examination robs us of the opportunity of demonstrating the accuracy of this conclusion, but there can be little doubt that it is substantially accurate. The record of the case as a whole illustrates several valuable clinical lessons. First, it emphasises the necessity for an ophthalmoscopic examination in any case where the symptoms, however slight these may be, are possible expressions of intra-cranial disease. Secondly, it shows that while such disease may produce so extreme a degree of optic neuritis as to lead to subsequent blindness, the other symptoms caused by the intra-cranial disease may be both few in number and slight in degree. In the third place, it illustrates the possible existence of optic neuritis with unimpaired vision. And yet again, it displays some of the possibilities attending the clinical history of an intra-cranial tumour, and suggests how these influence prognosis. The first conclusion to which many are disposed to come when they accept a diagnosis of intra-cranial tumour is that unless the tumour is syphilitic and can be removed by treatment, or is within the reach of the surgeon's knife, a rapid movement towards a fatal end is certain. Granted the accuracy of the diagnosis, the present case shows that such a result is by no means inevitable. The prognosis, therefore, in any case of intra-cranial disease must take into account the possibility of the tumour ceasing to grow. It must also, as is here illustrated, take into account the possibility that, even after a prolonged period of quiescence, the tumour may again become active. These facts have some bearing on the question of treatment. If, at least in some instances, an intra-cranial tumour tends to stay its growth, it is manifest that every effort should be made to encourage this tendency, and to keep the patient alive in the hope that this tendency will appear. Hence the necessity for early diagnosis, which, as seen in the present case, may be impossible unless the ophthalmoscope is used. Once recognised, it may be hoped that by the use of such remedies as potassium iodide and mercurial inunction the growth of the tumour may be restrained, at least in some instances. If these measures do not appear to be lessening the optic neuritis, it is a reasonable step to consider the advisability of trephining the skull in the hope that the reduction of intra-cranial pressure may have this result. For obviously, if a patient may recover (even if only for a time) from all the symptoms of cerebral tumour except the blindness consequent on post-neuritic optic atrophy, it becomes highly desirable to prevent this atrophy. And favourable influences in this direction may certainly be exerted by the means above suggested. Another consideration in reference to treatment arises from the risk that the tumour after a more or less prolonged period of quiescence may again become active. What circumstances determine such renewed activity it is difficult to say, but the risk has to be reckoned with. Perhaps such regular habits of life as conduce to a condition of good health may have some restraining influence; and in children and young adults in whom intra-cranial tumours are often tubercular, it is wise at frequent intervals to order a course of such remedies as cod liver oil and iodide of iron. A complete review of the case carries the conviction that, serious as a condition of intra-cranial tumour must necessarily be, there is a definite chance in some instances of the tumour ceasing to grow, and that, given early diagnosis and prompt treatment, the physician may do much to assist this tendency and also to preserve the patient from subsequent disastrous developments.

